# A calcified pelvic gossypiboma remaining clinically silent for 46 years and mimicking a degenerated uterine leiomyoma

**DOI:** 10.1093/jscr/rjag952

**Published:** 2026-10-22

**Authors:** Kenji Yorita, Jun Iwabu, Kimiko Nakatani

**Affiliations:** Department of Diagnostic Pathology, Japanese Red Cross Kochi Hospital, 1-4-63-11, Hadaminamimachi, Kochi City, Kochi 780-8562, Japan; Department of Surgery, Japanese Red Cross Kochi Hospital, 1-4-63-11, Hadaminamimachi, Kochi City, Kochi 780-8562, Japan; Department of Radiology, Japanese Red Cross Kochi Hospital, 1-4-63-11, Hadaminamimachi, Kochi City, Kochi 780-8562, Japan

**Keywords:** gossypiboma, surgical sponges, caesarean section, computed tomography, leiomyoma

## Abstract

Gossypiboma is a retained surgical sponge that may remain asymptomatic for decades and mimic pelvic tumours. We report a pelvic gossypiboma presenting as a calcified uterine mass 46 years after a caesarean section; to our knowledge, the longest reported interval to diagnosis of gossypiboma of any aetiology. A 74-year-old woman undergoing staging computed tomography for caecal adenocarcinoma was found to have a 4-cm pelvic mass with peripheral eggshell-like calcification adjacent to the uterine fundus, initially diagnosed as a degenerated leiomyoma. The mass, incarcerated between the caecum and uterus, was resected during ileocaecal resection and found to contain a calcified capsule enclosing foreign fibres; histology and polarized light microscopy confirmed retained surgical material. Unlike most reported gossypibomas, the lesion lacked the characteristic spongiform or reticulated calcification pattern. Gossypiboma should be considered in atypically calcified pelvic masses, regardless of the interval since surgery.

## Introduction

The term gossypiboma refers to a retained surgical sponge and is also known as textiloma or gauzeoma. It is a rare but important iatrogenic complication. Although most cases present with clinical symptoms and ~78% are diagnosed within the first year after surgery, some remain asymptomatic, with ~8% being diagnosed more than 5 years later [1]. After caesarean section specifically, the reported interval to diagnosis averages only 3.7 years, with the longest previously documented interval being 29 years [2]. In such long-standing cases, gossypiboma may present as a calcified or soft-tissue mass, thereby making the diagnosis challenging and often leading to misdiagnosis [3].

Here, we report the case of a 74-year-old woman with a history of a caesarean section ~46 years earlier who presented with a calcified pelvic mass initially suspected to be a degenerated uterine leiomyoma. As the mass was preoperatively diagnosed as a leiomyoma, its resection was planned concurrently with surgery for caecal cancer; subsequent histopathological examination unexpectedly confirmed the diagnosis of gossypiboma. This interval exceeds previously reported latencies after caesarean section and highlights the importance of considering gossypiboma in the differential diagnosis of calcified pelvic masses, regardless of how remote the surgical history.

## Case report

A 74-year-old woman underwent staging computed tomography (CT) for caecal adenocarcinoma, which incidentally revealed a 4-cm pelvic mass with peripheral eggshell-like calcification (Fig. 1A, arrow). The lesion showed no internal enhancement and was closely apposed to the uterine fundus (Fig. 1B, arrowheads), thereby leading to the initial radiological diagnosis of a degenerated uterine leiomyoma. The lesion lacked spongiform gas pattern or reticulated rind sign. Concurrent resection of the presumed leiomyoma was therefore planned alongside ileocaecal resection for the caecal adenocarcinoma.

**Figure 1:**
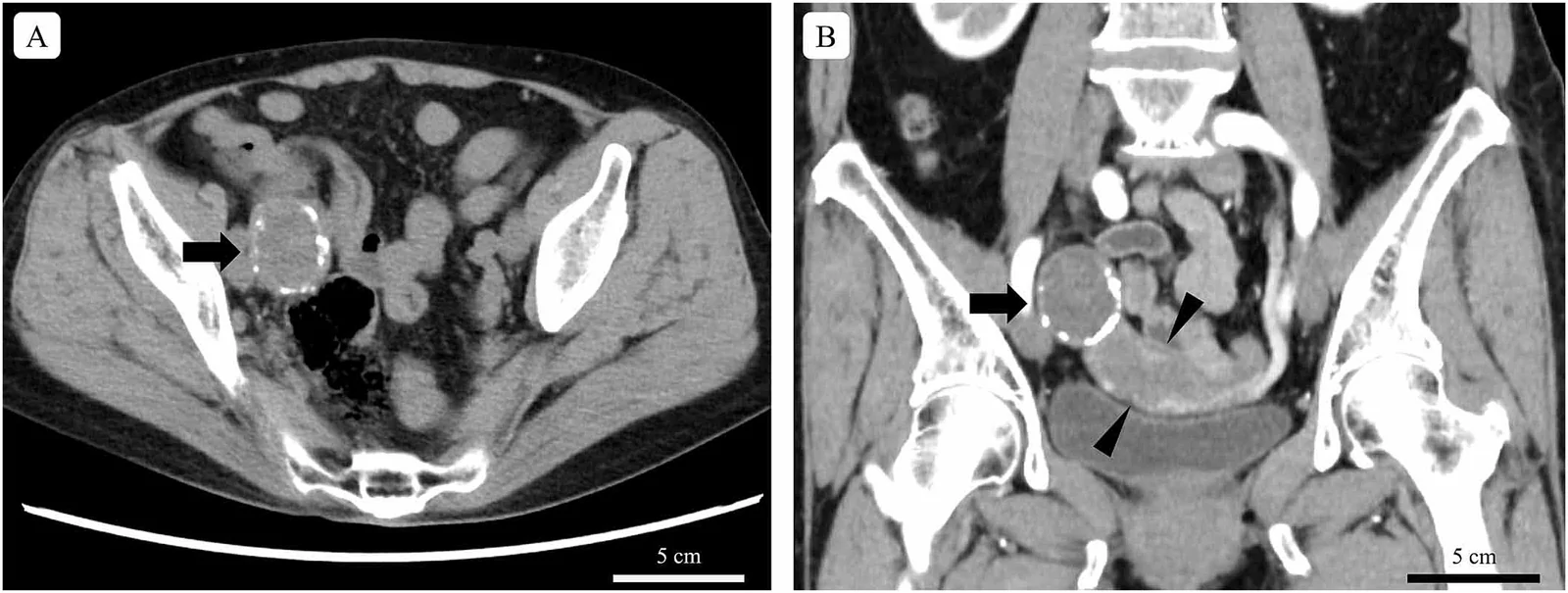
CT findings of a calcified pelvic gossypiboma. (A) Non-contrast axial CT showing a well-circumscribed pelvic mass with peripheral eggshell-like calcification (arrow). (B) Contrast-enhanced coronal CT reconstruction showing a non-enhancing mass (arrow) adjacent to the uterus (arrowheads).

During ileocaecal resection, the mass was found to be incarcerated between the caecum and the uterus. Upon excision, the calcified mass contained a compacted core of thread-like material. Stereomicroscopy revealed densely intertwined foreign fibres (Fig. 2A). Histopathological examination of the capsule showed hyalinized fibrosis with dystrophic calcification, whereas polarized light microscopy demonstrated birefringent filaments, confirming the presence of foreign material. In view of the patient’s history of a caesarean section ~46 years earlier, the lesion was ultimately diagnosed as gossypiboma.

**Figure 2:**
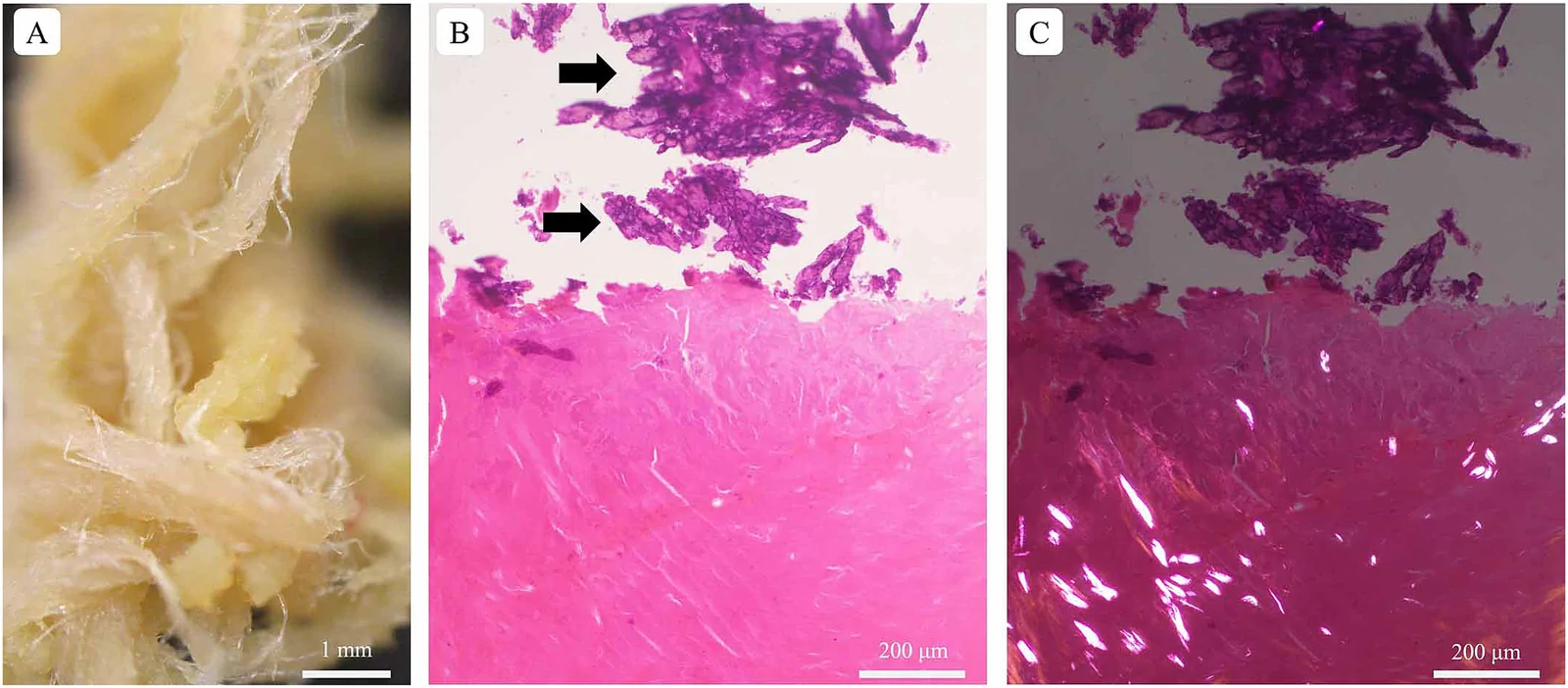
Stereomicroscopic and histological findings of a calcified pelvic gossypiboma. (A) Stereomicroscopy of the internal contents revealing a meshwork of exogenous fibres. (B and C) Histopathology of the capsule showing hyalinized fibrosis with dystrophic calcification (B, arrows; haematoxylin and eosin staining) and birefringent exogenous filaments under polarized light microscopy (C).

## Discussion

This case highlights that gossypiboma can remain clinically silent for decades and mimic common calcified pelvic masses, including uterine leiomyomas and adnexal tumours. Gossypibomas occur most frequently after caesarean section, hysterectomy, and emergency abdominal surgery, with only rare cases remaining asymptomatic for several decades [1].

To our knowledge, the 46-year interval in this case exceeds the longest previously reported latency specifically after caesarean section, which was 29 years in a recent literature review [2]. It also exceeds the longest intervals reported for gossypiboma of any aetiology that we could identify in the literature, including a spinal mass presenting 40 years after laminectomy [6], a paravesical foreign-body granuloma discovered 40 years after the inciting operation [7], and a soft-tissue mass at an iliac bone-graft donor site approximately four decades after cervical spine fusion [8]; a more recent report described a heterogeneous, non-calcified pelvic mass 38 years after hysterectomy [9]. To our knowledge, therefore, this case may represent the longest reported interval between the inciting surgery and diagnosis of gossypiboma in the literature. Calcification itself is uncommon among gossypibomas following caesarean section: in a review of 57 such cases, only one showed calcification on imaging, and the review did not describe any of the 57 cases—including that one—as having been mistaken for a uterine leiomyoma [2]. Our case, presenting as a calcified pelvic mass mimicking a degenerated uterine leiomyoma and lacking the spongiform gas pattern or reticulated rind sign, therefore adds a rare and diagnostically instructive example to this literature. Because caesarean section accounts for over one-fifth of deliveries worldwide, a substantial population of women carries a lifelong, often unrecognized risk of this diagnosis, underscoring the practical value of reporting atypical, non-classic presentations such as this one.

On CT, gossypibomas classically appear as a cystic lesion with a spongiform pattern containing gas bubbles [4]. Long-standing lesions may instead lose this pattern and show peripheral calcification, occasionally with a reticulated pattern along the gauze folds known as the calcified reticulated rind sign [5]. Notably, *in vitro* studies have shown that gas bubbles within surgical sponges are not significantly reduced within 6 months [4], yet none of the markedly long-standing cases reviewed here, each spanning four decades or more, showed a spongiform gas pattern: it was explicitly described as absent in the paravesical granuloma [7], and was likewise not evident in the iliac bone-graft-site mass [8]. Calcification, however, was inconsistent between these two cases: not evident on CT in the former [7], but present in the latter as discontinuous, eggshell-like peripheral calcification [8] that closely resembles the pattern seen in our patient rather than the calcified reticulated rind sign. Together with the present case, these observations suggest that although the spongiform gas pattern can persist over months, it may no longer be present after several decades, and that peripheral calcification, when it does occur in such long-standing cases, does not always follow a reticulated pattern; given the small number of comparable cases, this observation should be interpreted with caution.

In women with a calcified pelvic mass, the differential diagnosis includes uterine leiomyomas, mature cystic teratomas, and ovarian cystic neoplasms [10, 11]. Although our patient had a remote history of caesarean section, retrospective review of the preoperative CT images failed to demonstrate the characteristic reticulated calcification, making a preoperative diagnosis difficult. Therefore, definitive diagnosis required histopathological examination of the resected specimen. Accordingly, this case underscores the importance of considering gossypiboma in the differential diagnosis of calcified pelvic masses in patients with a history of surgery, regardless of the elapsed time.
